# Polymethyl methacrylate does not adversely affect the osteogenic potential of human adipose stem cells or primary osteoblasts

**DOI:** 10.1002/jbm.b.34501

**Published:** 2019-10-24

**Authors:** Angela P. Bastidas‐Coral, Astrid D. Bakker, Cornelis J. Kleverlaan, Jolanda M. A. Hogervorst, Jenneke Klein‐Nulend, Tymour Forouzanfar

**Affiliations:** ^1^ Department of Oral Cell Biology, Academic Centre for Dentistry Amsterdam (ACTA) University of Amsterdam and Vrije Universiteit Amsterdam, Amsterdam Movement Sciences Amsterdam The Netherlands; ^2^ Department of Dental Materials Science, Academic Centre for Dentistry Amsterdam (ACTA) University of Amsterdam and Vrije Universiteit Amsterdam, Amsterdam Movement Sciences Amsterdam The Netherlands; ^3^ Department of Oral and Maxillofacial Surgery/Oral Pathology Amsterdam University Medical Centers (Amsterdam UMC)‐location VUmc/Academic Centre for Dentistry Amsterdam (ACTA), Amsterdam Movement Sciences Amsterdam The Netherlands

**Keywords:** adipose stem cells, cranial bone defect, cytotoxicity, osteogenesis, polymethyl methacrylate

## Abstract

Custom‐made polymethyl methacrylate (PMMA) bone cement is used to treat cranial bone defects but whether it is cytotoxic is still unsure. Possible PMMA‐induced adverse effects in vivo affect mesenchymal stem cells and osteoblasts at the implant site. We aimed to investigate whether PMMA affects osteogenic and osteoclast activation potential of human mesenchymal stem cells and/or osteoblasts. Immediately after polymerization, PMMA was added to cultured human adipose stem cells (hASCs) or human osteoblasts (hOBs). Medium lactate dehydrogenase was measured (day 1), metabolic activity, proliferation, osteogenic and osteoclast‐activation marker expression (day 1 and 7), and mineralization (day 14). PMMA did not affect lactate dehydrogenase, KI67 gene expression, or metabolic activity in hASCs and hOBs. PMMA transiently decreased DNA content in hOBs only. PMMA increased COL1 gene expression in hASCs, but decreased RUNX2 in hOBs. PMMA did not affect osteocalcin or alkaline phosphatase (ALP) expression, ALP activity, or mineralization. Only in hOBs, PMMA decreased RANKL/OPG ratio. In conclusion, PMMA is not cytotoxic and does not adversely affect the osteogenic potential of hASCs or hOBs. Moreover, PMMA does not enhance production of osteoclast factors by hASCs and hOBs in vitro. Therefore, PMMA bone cement seems highly suitable to treat patients with cranial bone defects.

## 1 INTRODUCTION

Cranial defects can result from pathological and/or traumatic insults. Cranioplasty is usually performed to restore the protective function of the skull, with a secondary goal of restoring cranial aesthetics. Different materials have been used for the treatment of cranial defects, for example, autologous bone grafts, titanium implants, polyether‐ether‐ketone, and polymethyl methacrylate (PMMA), the latter being the most widely used alloplastic material due to its high mechanical strength and relatively low cost (Kim et al., 2012). PMMA bone cement is assumed to be a non‐degradable and biocompatible implant material that can be prefabricated, or even molded intraoperatively, by mixing a liquid monomer methyl methacrylate (MMA) and a powder polymer (PMMA) (Abdo Filho, Oliveira, Lourenco Neto, Gurgel, & Abdo, 2011; Dayashankara Rao, Malhotra, Batra, & Kukreja, 2011). Thereafter, time is required to allow the resulting PMMA material to polymerize, during which time monomer linking and subsequent formation of solid polymer occur. The MMA monomer is known to be cytotoxic (Ansteinsson, Kopperud, Morisbak, & Samuelsen, 2013; Hattori et al., 2008; Jinno et al., 2006; Schweikl, Schmalz, & Spruss, 2001). Although the mixing and initial polymerization process occurs outside the implantation site, it is unknown whether PMMA that has not completely polymerized has adverse effects when it comes into contact with the bone tissue at the implant site.

The use of PMMA has two main disadvantages, that is, the highly exothermic reaction during the polymerization process, and the potential release of non‐reacted monomers, which may cause damage to the surrounding bone tissue after PMMA implantation (Dunne & Orr, 2002). Aseptic loosening of PMMA implants caused by monomer‐mediated bone damage has been reported previously, as well as neurotoxicity due to the monomer, and lack of osteointegration, because of the bio‐inert properties of the material (Dahl, Garvik, & Lyberg, 1994; Heini & Berlemann, 2001; Jaeblon, 2010). Although implant failure has been reported to occur, the potential adverse effect of PMMA bone cement on the surrounding bone tissue has to be weighed against the advantageous high mechanical strength and stability provided by the material.

Mesenchymal stem cells (MSCs) and osteoblasts present within the tissue surrounding a bone defect are potentially exposed to cytotoxic factors leaching from the implanted PMMA. In vivo, MSCs migrate to the implant site, where they differentiate into an osteoblastic phenotype (Verrier et al., 2012). PMMA particles have been shown to inhibit osteoblast proliferation and collagen synthesis which may result in reduced periprosthetic bone formation, whereas PMMA particles stimulate osteocalcin and IL‐6 synthesis, known to stimulate bone resorption (Ishida & Amano, 2004; Kudo et al., 2003; Zambonin, Colucci, Cantatore, & Grano, 1998). In addition, in a murine bone marrow cell culture in vitro, PMMA particles added to mature osteoclasts result in an increase in the number of TRAP‐positive cells which persisted over a longer time period, and increased bone resorption (Zhang et al., 2008). Thus, PMMA may affect osteoblast and osteoclast activity in a way that could contribute to peri‐prosthetic osteolysis.

Whether PMMA as a bulk material can adversely affect cells present in the bone tissue surrounding the implant, for example, through the leaching of non‐reacted monomers, is still unknown. Knowledge about possible adverse effects is highly relevant as implanted PMMA material is in direct contact with the bone tissue where MSCs and osteoblasts are present. Therefore, the aim of our study was to investigate whether PMMA affects the osteogenic potential, and the expression of signaling molecules that regulate osteoclast activity in hASCs and/or osteoblasts. We measured lactate dehydrogenase (LDH) levels, metabolic activity, proliferation, and expression of osteogenic differentiation and osteoclast‐activation markers by both cell types, in comparison to control cells without PMMA. We hypothesized that PMMA is cytotoxic and adversely affects metabolic activity, proliferation, osteogenic potential, as well as osteoclast activation potential by hASCs and hOBs.

## 2 MATERIALS AND METHODS

### 2.1 Preparation of PMMA

Commercially available PMMA was obtained from two companies (PMMA bone cement, Antibiotic Simplex®; Stryker Orthopaedics, Mahwah, New Jersey, and Rapid Simplified; Vertex dental BV, Zeist, The Netherlands). In our study we used PMMA samples polymerized at distinct temperatures and for different time periods, that is, PMMA polymerized at room temperature for 15 min, at 60°C for 5 min, or at 100°C for 20 min. Increasing polymerization temperature of PMMA has been shown to decrease residual monomer content of polymers, and to improve PMMA compatibility (Vallittu, Ruyter, & Buykuilmaz, 1998). For experiments, PMMA was prepared according to the manufacturer's instructions. Briefly, the liquid monomer (methylmethacrylate‐styrene) was mixed with the powder polymer PMMA at room temperature, and molds were filled to obtain a disk‐shaped material (diameter: 1 cm, height: 0.2 cm). PMMA samples were allowed to polymerize at room temperature for 15 min, at 60°C for 5 min, or at 100°C for 20 min.

### 2.2 TheraCal LC®

TheraCal LC® (Bisco, Schaumburg, Illinois) consists of tri‐calcium silicate particles in a hydrophilic monomer such as hydroxyethyl methacrylate (HEMA) and polyethylene glycol dimethacrylate (PEGDMA) (Cannon, Martin, Suh, & Yin, n.d.). These monomers have been reported to be cytotoxic (Chang et al., 2005; Orimoto et al., 2013). Therefore, we included TheraCal LC® in our experiments as an internal positive control. TheraCal LC® was used according to the manufacturer's instructions. Briefly, TheraCal LC® consisting of a single paste was placed into a mold to obtain a disk‐shaped material (diameter: 1 cm, height: 0.2 cm), and light cured using an UV‐lamp (3 M ESPE Elipar™ S10, St. Paul, Minnesota).

### 2.3 Isolation and culture of hASCs

Subcutaneous adipose tissue samples were harvested from abdominal wall resections of five healthy female donors (age range: 33–54 years, mean: 47 years), who underwent elective plastic surgery at the Tergooi Hospital Hilversum and a clinic in Bilthoven, The Netherlands. The Ethical Review Board of the VU University Medical Center, Amsterdam, The Netherlands, approved the protocol (number 2016/105) and informed consent was obtained from all patients. Isolation, characterization and osteogenic differentiation capacity of hASCs has been previously reported by our group (Varma et al., 2007). Adipose tissue represents a promising source of MSCs, as liposuction can be performed with minimal patient discomfort and yields higher numbers of MSCs than bone marrow. In addition, a previous study by our group has demonstrated the safety, feasibility, and efficacy of the use of hASCs in human maxillary sinus floor elevation, and the pro‐angiogenic effect of ASC‐containing stromal vascular fraction (Farre‐Guasch et al., 2018).

Briefly, the collected adipose tissue was cut in small pieces and enzymatically digested with 0.1% collagenase A (Roche Diagnostics GmbH, Mannheim, Germany) in phosphate‐buffered saline (PBS) containing 1% bovine serum albumin (Roche Diagnostics GmbH) under continuous shaking conditions for 45 min at 37°C. Next, a Ficoll® density‐centrifugation step (Lymphoprep™; 1,000 g, 20 min, *ρ* = 1.077 g/mL Ficoll®, osmolarity 280 ± 15 mOsm; Axis‐Shield, Oslo, Norway) was performed, and the cell containing interface was harvested and resuspended in Dulbecco's modified Eagle's medium (DMEM; LifeTechnologies™ Europe BV, Bleiswijk, The Netherlands). Finally, hASCs were counted and stored in liquid nitrogen. Cryopreserved hASCs from the different donors were pooled and cultured in α‐Minimum Essential Medium (α‐MEM; Gibco, Life Technologies, Waltham, Massachusetts) with 1% penicillin, streptomycin, and fungizone (PSF; Sigma, Saint Louis, Missouri), 10 IU/mL heparin (LEO Pharma A/S, Ballerup, Denmark) and 5% human platelet lysate (PL) at 37°C in a humidified atmosphere of 5% CO_2_ in air. At near confluency (90%), hASCs were harvested by adding 0.25% trypsin (Gibco, Invitrogen, Waltham, Massachusetts), and 0.1% ethylene‐diamine‐tetraacetic acid (EDTA) (Merck, Darmstadt, Germany) in PBS, and stored in liquid nitrogen until further use. For experiments, hASCs stored in liquid nitrogen were thawed and seeded at 0.5 × 10^6^ cells in T‐175 cm^2^ culture flasks (Greiner Bio‐One, Kremsmuenster, Austria) in αMEM containing 1% PSF, 10 IU/mL heparin, and 2% human PL in a humidified atmosphere of 5% CO_2_ in air at 37°C. In all experiments, hASCs at passage 3 (P3) were used. Medium was changed every 3 days.

### 2.4 Isolation and culture of hOBs

Surgical waste human bone from hip and knee was obtained from two healthy male donors (age: 60 and 74 years), after surgery performed at the VU University Medical Center, Amsterdam, The Netherlands. This in agreement with The Ethical Review Board of the VU Medical Center, Amsterdam, The Netherlands, under protocol number 2016/105. Bone cell cultures were performed as described earlier (Klein‐Nulend et al., 2002). Briefly, bone biopsies were cut into small pieces and treated with collagenase solution (type II, Worthington, Lakewood, NJ, USA) under continuous shaking conditions for 2 hr at 37°C. The small bone fragments were placed into a T‐75 culture flask and cultured in Dulbecco's Modified Eagle Medium *(*DMEM; Gibco®, Life Technologies, Waltham, Massachusetts), with 1% PSF, 10 IU/mL heparin, and 5% human PL at 37°C in 5% CO_2_. The medium was changed every 3 days. After reaching 90% confluency, hOBs at passage 1 (P1) were counted and stored in liquid nitrogen until further use. For all experiments, a pool of cells obtained from two donors at P2 was used.

### 2.5 Platelet lysate

Pooled platelet products from five donors were obtained from the Bloodbank Sanquin (Sanquin, Amsterdam, The Netherlands) and contained approximately 1 × 10^9^ platelets per ml (Prins et al., 2009). PL was obtained by lysing the platelets through temperature shock at −80°C. Before usage PL was thawed and centrifuged at 600*g* for 10 min to eliminate remaining platelet fragments. The supernatant was added at 2% (v/v) to the medium.

### 2.6 Culture of hASCs and hOBs with PMMA

hASCs and hOBs were seeded into 12‐well plates at 1x10^4^ cells/cm^2^, and cultured in αMEM containing 1% PSF, 10 IU/mL heparin, and 2% human PL (hASCs) or 5% human PL (hOBs) in a humidified atmosphere of 5% CO_2_ in air at 37°C. hASCs and hOBs were allowed to attach during 24 hr before adding PMMA material. The PMMA was placed in a *transwell* insert (pore size, 3.0 μm; Greiner Bio‐one, Alphen aan den Rijn, The Netherlands). Cells cultured without PMMA were used as control. After 24 h, medium was replaced with osteogenic medium containing α‐MEM (hASCs) or DMEM (hOBs), supplemented with 1% PSF, 10 IU/mL heparin, and 2% (hASCs) or 5% (hOBs) human PL, 50 μM ascorbic acid‐2‐phosphate (vitamin C; Sigma, Saint Louis, MO, USA), 5 mM β‐glycerophosphate (βGP) (Sigma) and 10 nM 1,25‐(OH)_2_‐vitamin D_3_) (Sigma). The medium was refreshed every 3 days. hASCs and hOBs were exposed to PMMA up to day 14 of culture.

### 2.7 Cytotoxicity assay

The potential cytotoxic effect of PMMA on hASCs and/or hOBs was assessed by measuring LDH (Roche, Mannheim, Germany). After 1 day of culture of hASCs and hOBs with PMMA, supernatants were collected and incubated with reaction mixture for 30 min at room temperature. The LDH catalyzed conversion results in reduction of tetrazolium salt to formazan, which was measured at 490 nm in a Synergy HT® spectrophotometer (BioTek Instruments, Winooski, Vermont). The amount of LDH release is proportional to the number of lysed cells. Cytotoxicity of PMMA on hASCs and hOBs expressed as percent LDH activity was determined relative to controls as described by the manufacturer. Cells treated with 1% Triton‐X100 were used as positive (maximum) LDH release control.

### 2.8 Metabolic activity assay

Metabolic activity of hASCs and hOBs cultured with PMMA was assessed by using AlamarBlue™ Cell viability reagent (Invitrogen, Rockford, IL, USA). Metabolic activity of hASCs and hOBs was analyzed at 1 and 7 days. Cells were incubated with medium containing 10% AlamarBlue for 4 hr at 37°C. Following incubation, 100 μL supernatant was transferred into a black 96‐well plate and fluorescence was measured at 530–560 nm wavelength in a Synergy HT® spectrophotometer. Medium without cells containing 10% AlamarBlue was placed in an autoclave container, and used as positive control of 100% chemically reduced AlamarBlue solution.

### 2.9 DNA content quantification

hASCs and hOBs cultured for 1 or 7 days with PMMA were washed with PBS, and lysis buffer was added. DNA content as a measure of total cell number was determined using the Cyquant Cell Proliferation Assay Kit (Molecular Probes, Leiden, The Netherlands). Absorption was read at 485 nm excitation and 528 nm emission with a Synergy HT® spectrophotometer.

### 2.10 RNA isolation and real‐time PCR

hASCs and hOBs RNA isolation was performed using the RNeasy Mini Kit (74,106, Qiagen, Venlo, The Netherlands) according to the manufacturer's instructions. RNA concentration and quality was measured using a Synergy HT® spectrophotometer. RNA was reverse‐transcribed to cDNA using a RevertAid™ First Strand cDNA Synthesis Kit (Fermentas, St. Leon‐Rot, Germany) according to manufacturer's instructions. The obtained cDNA was diluted to a final concentration of 2 ng/μl and stored at −20°C until further use. Quantitative real‐time PCR (qPCR) was performed using the SYBR® Green I Mastermix (Roche Diagnostics, Mannheim, Germany) in a LightCycler® 480 (Roche Diagnostics, Basel, Switzerland). Every PCR reaction was prepared with 4 μL cDNA, 0.5 μL forward primer (1 μM), 0.5 μL reverse primer (1 μM), 5 μL LightCycler® 480 SYBR® Green I Mastermix (Roche Diagnostics, Mannheim, Germany) in a final volume of 10 μL. qPCR conditions for all genes were as follows: 10 min pre‐incubation at 95°C, followed by 45 cycles of amplification at 95°C for 10 s, 56°C for 5 s, 72°C for 10 s, and 78°C for 5 s, after which melting curve analysis was performed. With LightCycler® (version 1.2), crossing points were assessed and plotted versus the serial dilution of known concentrations of the internal standard. mRNA preparations of hASCs and hOBs were used as a reference and internal standard in each assay. PCR efficiency (E) was obtained by using the formula E = 10^–1/slope^. Data were used only if E = 1.85–2.00. For gene expression analysis, the values of target gene expression were normalized to reference genes GUSB, TBP, and YWHAZ based on Bestkeeper software (Pfaffl, Tichopad, Prgomet, & Neuvians, 2004). The relative expression of a gene of interest was calculated in relation to the reference gene on basis of the crossing point (Cp) deviation (delta Cp). qPCR was used to assess expression of the following genes: KI67, RUNX2, COL1, osteocalcin, alkaline phosphatase (ALP), OPG, and RANKL. All primers used were from Life Technologies (Invitrogen, Thermo fisher Scientific, Eugene, OR, USA). Primer sequences are listed in Table 1.

**Table 1:** List of primer sequences used for analysis of proliferation, expression of osteogenic markers by hASCs and hOBs, and signaling markers for osteoclast differentiation by PCR

Target gene (human)	Oligonucleotide sequences		Accession number	Primer efficiency (E)
*GUSB*	Forward	5’ CGCACAAGAGTGGTGCTGAG 3’	NM_000181	1.89
	Reverse	5’ GGAGGTGTCAGTCAGGTATT 3’		
*TBP*	Forward	5’ GGTCTGGGAAAATGGTGTGC 3’	NM_003194	1.90
	Reverse	5’ GCTGGAAAACCCAACTTCTG 3’		
*YWHAZ*	Forward	5’ GATGAAGCCATTGCTGAACTTG 3’	NM_003406	1.94
	Reverse	5’ CTATTTGTGGGACAGCATGGA 3’		
*KI67*	Forward	5’ CCCTCAGCAAGCCTGAGAA 3’	NM_002417.4	1.90
	Reverse	5’ AGAGGCGTATTAGGAGGCAAG 3’		
*RUNX2*	Forward	5’ ATGGTTCATTCGCCTCAC 3’	NR_103532	1.91
	Reverse	5’ ACTGCTTGCAGCCTTAAAT 3’		
*COL1*	Forward	5’ TCCGGCTCCTGCTCCTCTTA 3’	NM_000088	2.0
	Reverse	5’ GGCCAGTGTCTCCCTTG 3’		
*OC*	Forward	5’ AGCCACCGAGACACCATGAGA 3’	NM_000711	1.93
	Reverse	5’ CTCCTGAAAGCCGATGTGGTC 3’		
*ALP*	Forward	5’ GCTTCAAACCGAGATACAAGCA 3’	BC021289	1.92
	Reverse	5’ GCTCGAAGAGACCCAATAGGTAGT 3’		
*OPG*	Forward	5’ TGGAATAGATGTTACCCTGTGTG 3’	NM_002546	1.95
	Reverse	5’ GCTGCTCGAAGGTGAGGTTA 3’		
*RANKL*	Forward	5’ CATCCCATCTGGTTCCCATAA 3’	NM_003701	1.95
	Reverse	5’ GCCCAACCCCGATCATG 3’		

Abbreviations: ALP, alkaline phosphatase; COL1, collagen‐type 1; GUSB: β‐glucuronidase; KI67, proliferation marker; OC, osteocalcin; OPG, osteoprotegerin; RANKL, receptor activator of nuclear factor‐κB ligand; RUNX2, runt‐related transcription factor‐2; TBP, TATA‐box binding protein; YWHAZ, tyrosine 3‐monooxygenase/tryptophan 5‐monooxygenase activation protein zeta.

### 2.11 ALP activity

hASC and hOBs cultured for 1 or 7 days with PMMA were lysed with 500 μL lysis buffer and stored at −20°C prior to use. ALP activity was measured in the cell lysate using 4‐nitrophenyl phosphate disodium salt (Merck, Darmstadt, Germany) at pH 10.3 as a substrate, according to the manufacturer's instructions. The absorbance was read at 405 nm with a Synergy HT® spectrophotometer. ALP activity was quantified against a standard curve of 4‐nitrophenol solution and expressed as μmol 4‐nitrophenol per ng DNA.

### 2.12 Mineralization

Matrix mineralization was analyzed by alizarin red staining of hASCs or hOBs cultured with or without PMMA at day 14, by using 2% Alizarin Red S (Sigma‐Aldrich, St Louis, MO, USA) in water at pH 4.3. Cells were fixed with 4% formaldehyde for 15 min and rinsed with deionized water. Five‐hundred microliter per well alizarin red solution was added for 30 min. Differentiated osteoblasts show calcium deposition (mineralization) visible as bright red nodules. Quantification of mineralized matrix was performed using ImageJ software (ImageJ 1.49v, USA) as previously described by our group (Shah et al., 2016).

### 2.13 Statistical analysis

Values are presented as mean ± SEM. In total three independent experiments were performed in duplicate (n = 3). Statistical significance was determined using analysis of variance (ANOVA), with Dunnett's multiple comparison test to compare against the control group. A P‐value <0.05 was considered significant. Statistical analysis was performed using GraphPad Prism 6 (GraphPad Software, San Diego, California).

## 3 RESULTS

### 3.1 Metabolic activity and proliferation of hASCs and hOBs

The effect of polymerized PMMA on LDH release (indicator of cell death and lysis), AlamarBlue metabolic activity, DNA content (indicating total cell number), and KI67 gene expression (proliferation marker), were quantified to determine possible cytotoxic effects on hASCs and/or hOBs.

**Figure 1:**
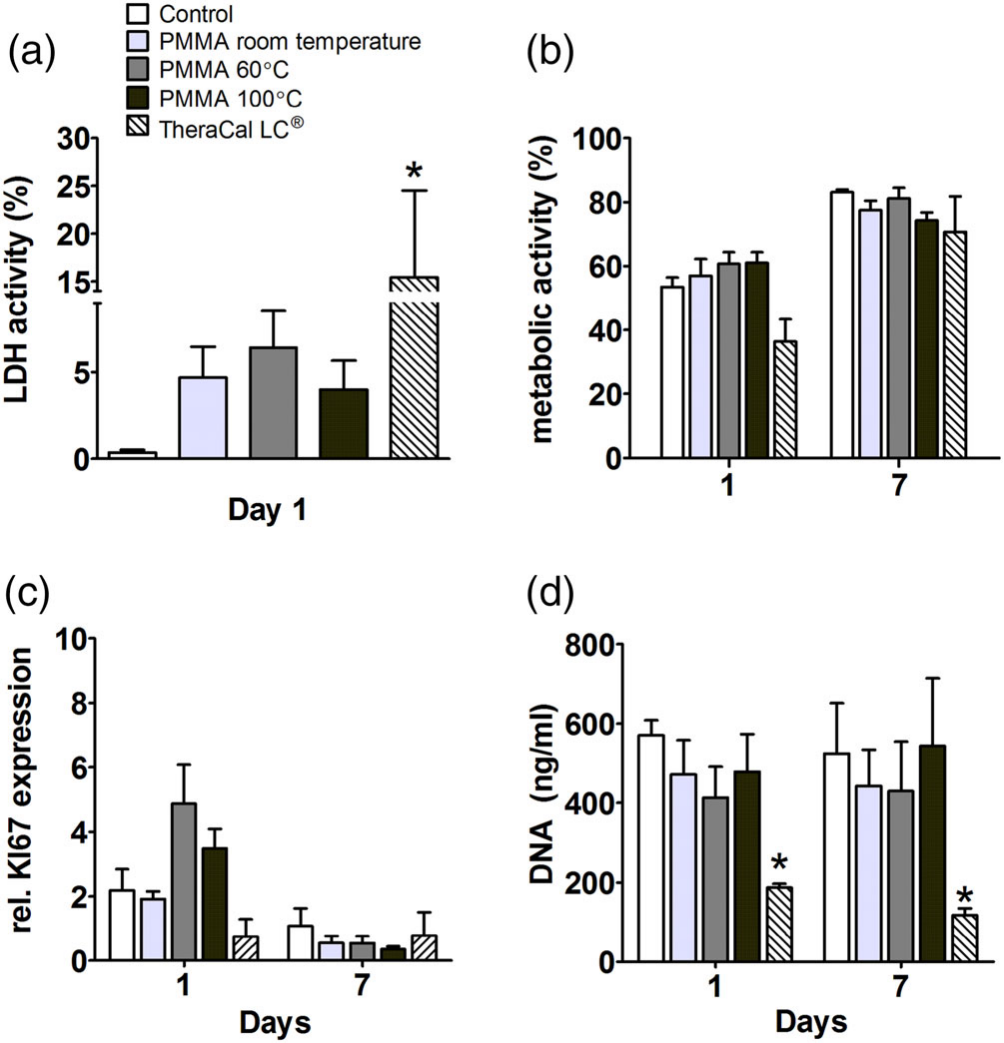
Effect of polymethyl methacrylate (PMMA) on lactate dehydrogenase (LDH) activity, metabolic activity, *KI67* gene expression, and DNA content in human adipose stem cells (hASCs). hASCs were cultured with PMMA polymerized at room temperature, 60°C, or 100°C, and with TheraCal LC® used as positive control for cytotoxicity. (a) LDH activity at day 1. (b) Metabolic activity at day 1 and 7. (c) Gene expression of proliferation marker *KI67* at day 1 and 7. (d) DNA content at day 1 and 7. Values are mean ± SEM of duplicate cultures from 3 independent experiments (*n* = 3) using hASCs obtained from five donors. *Significant effect of PMMA, *p* < .05

TheraCal LC®, the internal positive control for cytotoxicity, increased LDH release by 15‐fold at day 1 (Figure 1a), it did not affect metabolic activity in hASCs (Figure 1b) or KI67 gene expression (Figure 1c), but it decreased DNA content by threefold at day 1 and 7 (Figure 1d). At day 1, none of the PMMA polymerized at distinct temperatures (room temperature, 60°C, and 100°C), affected LDH release by hASCs (Figure 1a). PMMA also did not affect metabolic activity, expression of the proliferation marker KI67, or DNA content in hASCs at day 1 or 7 (Figure 1b–d).

**Figure 2:**
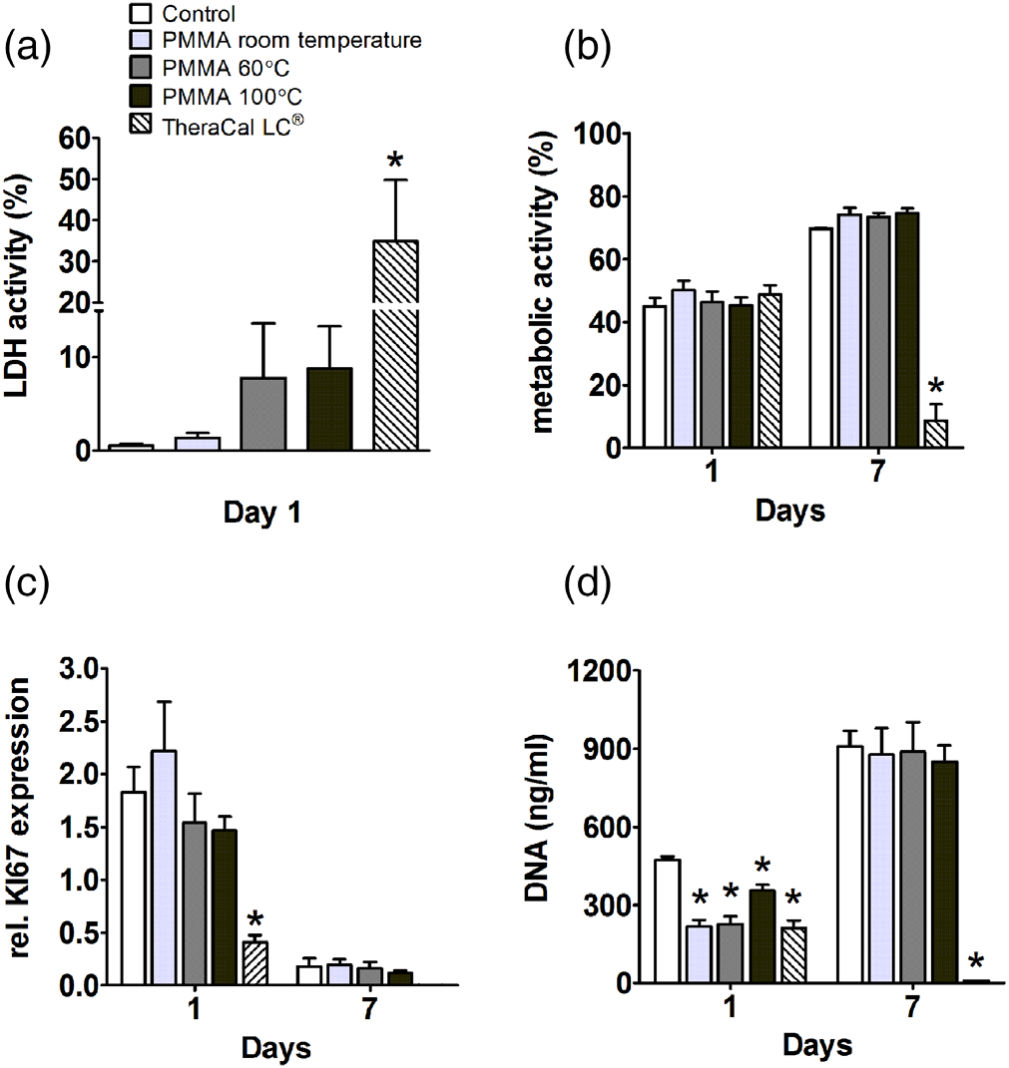
Effect of polymethyl methacrylate (PMMA) on lactate dehydrogenase (LDH) activity, metabolic activity, *KI67* gene expression, and DNA content in human osteoblasts (hOBs). hOBs were cultured with PMMA polymerized at room temperature, 60°C, or 100°C, or with TheraCal LC® used as a positive control for cytotoxicity. (a) LDH activity at day 1. (b) Metabolic activity at day 1 and 7. (c) Expression of proliferation marker *KI67* at day 1 and 7. (d) DNA content at day 1 and 7. Values are mean ± SEM of duplicate cultures from three independent experiments (*n* = 3) using bone cells obtained from two donors *Significant effect of PMMA, *p* < .05

In hOBs, TheraCal LC® increased LDH release by 34‐fold at day 1 (Figure 2a), and decreased metabolic activity by eightfold at day 7 (Figure 2b), KI67 gene expression by fourfold at day 1 (Figure 2c), as well as DNA content at day 1 and 7 (Figure 2d). PMMA, polymerized at distinct temperatures, did not cause cell lysis of hOBs measured by LDH release (Figure 2a). The metabolic activity of hOBs cultured with PMMA, polymerized at distinct temperatures, was not decreased at day 1 or 7 (Figure 2b). Although KI67 gene expression was similar in PMMA polymerized at distinct temperatures in hOBs (Figure 2c), DNA content decreased by 1.3‐ to 2.2‐fold in all groups compared to control cultures without PMMA at day 1 (Figure 2d). Taken together, PMMA polymerized at distinct temperatures, decreased DNA content in hOBs, but not hASCs. PMMA, polymerized at distinct temperatures, did not affect LDH release, metabolic activity of KI67 gene expression in hASCs or hOBs. Next, we analyzed whether PMMA did affect the osteogenic differentiation of hASCs and hOBs.

### 3.2 Expression of osteogenic markers by hASCs and hOBs

**Figure 3:**
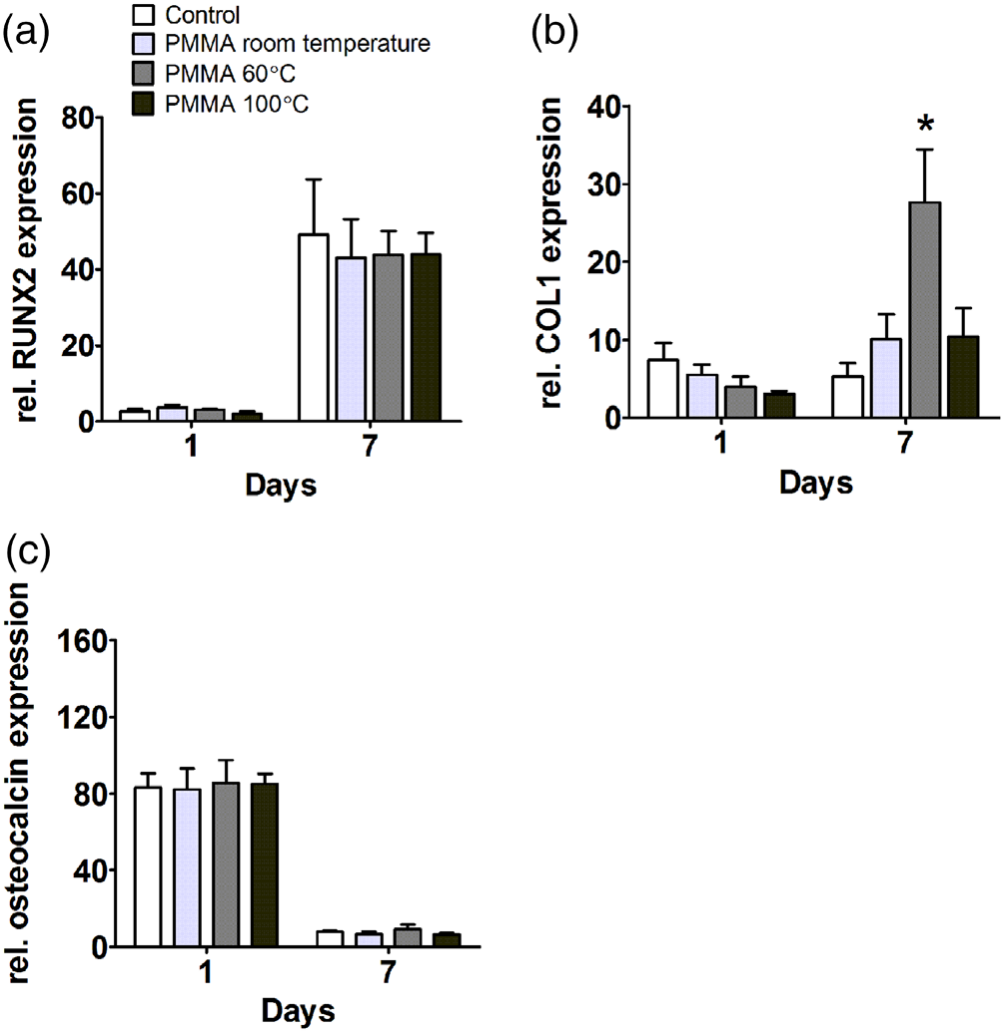
Effect of polymethyl methacrylate (PMMA) on osteogenic differentiation of human adipose stem cells (hASCs). hASCs were cultured with PMMA polymerized at room temperature, 60°C, or 100°C. (a) *RUNX2* gene expression at day 1 and 7. (b) *COL1* gene expression at day 1 and 7. (c) Osteocalcin gene expression at day 1 and 7. Values are mean ± SEM of duplicate cultures from three independent experiments (*n* = 3) using hASCs obtained from five donors. *Significant effect of PMMA, *p* < .05

The effect of PMMA on the osteogenic differentiation potential of hASCs and expression of osteogenic markers by hOBs was assessed by measuring gene expression of the early osteogenic differentiation markers RUNX2 and COL1, and the late marker osteocalcin. PMMA polymerized at room temperature and at 100°C did not affect the expression of RUNX2, COL1, or osteocalcin at day 1 and 7 in hASCs (Figure 3a–c). Only PMMA polymerized at 60°C enhanced COL1 gene expression by fivefold at day 7 (Figure 3b).

**Figure 4:**
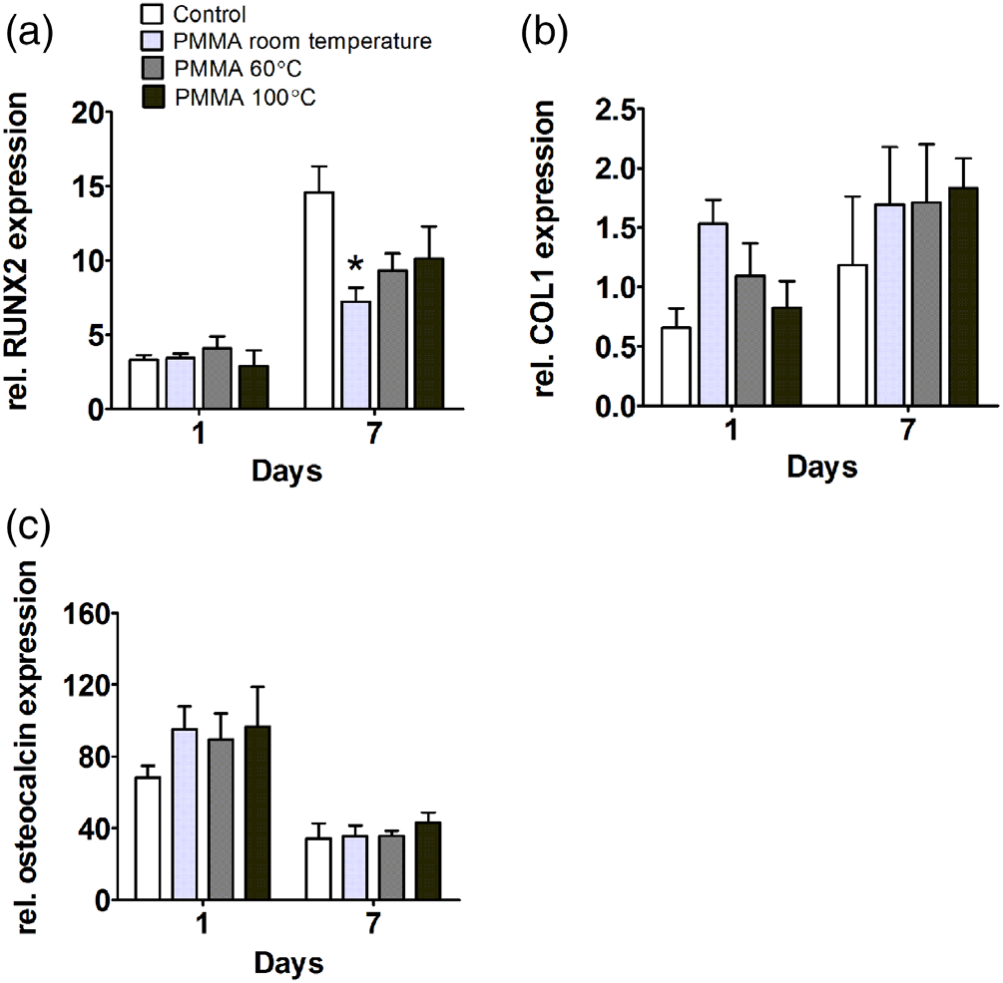
Effect of polymethyl methacrylate (PMMA) on the expression of osteogenic markers by human osteoblasts (hOBs). hOBs were cultured with PMMA polymerized at room temperature, 60°C, or 100°C. (a) *RUNX2* gene expression at day 1 and 7. (b) *COL1* gene expression at day 1 and 7. (c) Osteocalcin gene expression at day 1 and 7. Values are mean ± SEM of duplicate cultures from three independent experiments (*n* = 3) using bone cells obtained from two donors. *Significant effect of PMMA, *p* < .05

In hOBs, PMMA polymerized at room temperature decreased RUNX2 gene expression by twofold at day 7 (Figure 4a). PMMA polymerized at the other temperatures did not affect RUNX2, COL1, or osteocalcin expression either at day 1 and 7 (Figure 4a–c).

### 3.3 ALP activity and mineralization of hASCs and hOBs

**Figure 5:**
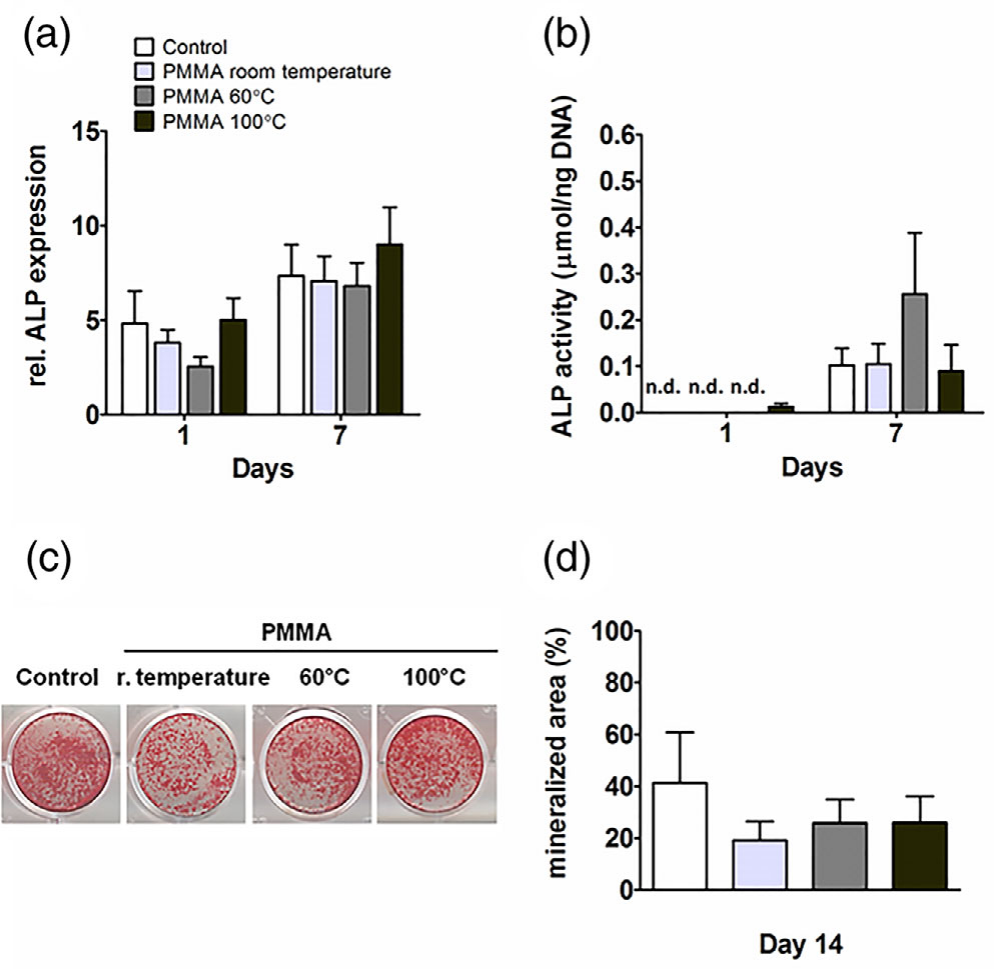
Effect of polymethyl methacrylate (PMMA) on ALP gene expression, ALP activity, and mineralization *in* human adipose stem cells (hASCs). hASCs were cultured with PMMA polymerized at room temperature, at 60°C, or at 100°C. (a) ALP gene expression at day 1 and 7. (b) ALP activity at day 1 and 7. (c) Mineralization at day 14. (d) Quantification of mineralized matrix at day 14. Values are mean ± SEM of duplicate cultures from three independent experiments (*n* = 3) using hASCs obtained from five donors. *Significant effect of PMMA, *p* < .05

An important marker of osteogenic differentiation is the effector protein ALP, which is an enzyme responsible for the mineralization of the extracellular matrix. Both mRNA and ALP activity were analyzed in hASCs and hOBs at day 1 and 7. PMMA, polymerized at distinct temperatures, did not decrease ALP gene expression by hASCs at day 1 and 7 (Figure 5a). ALP activity was not detectable in hASCs at day 1. No differences were observed between ALP activity in hASCs with PMMA, polymerized at distinct temperatures, at day 1 and 7 (Figure 5b). Matrix mineralization was analyzed using alizarin red staining of calcium deposition in hASCs and hOBs at day 14. Matrix mineralization of hASCs was not affected by PMMA at day 14 (Figure 5c). Quantification of the mineralized matrix showed that PMMA did not inhibit hASCs mineral deposition (Figure 5d).

**Figure 6:**
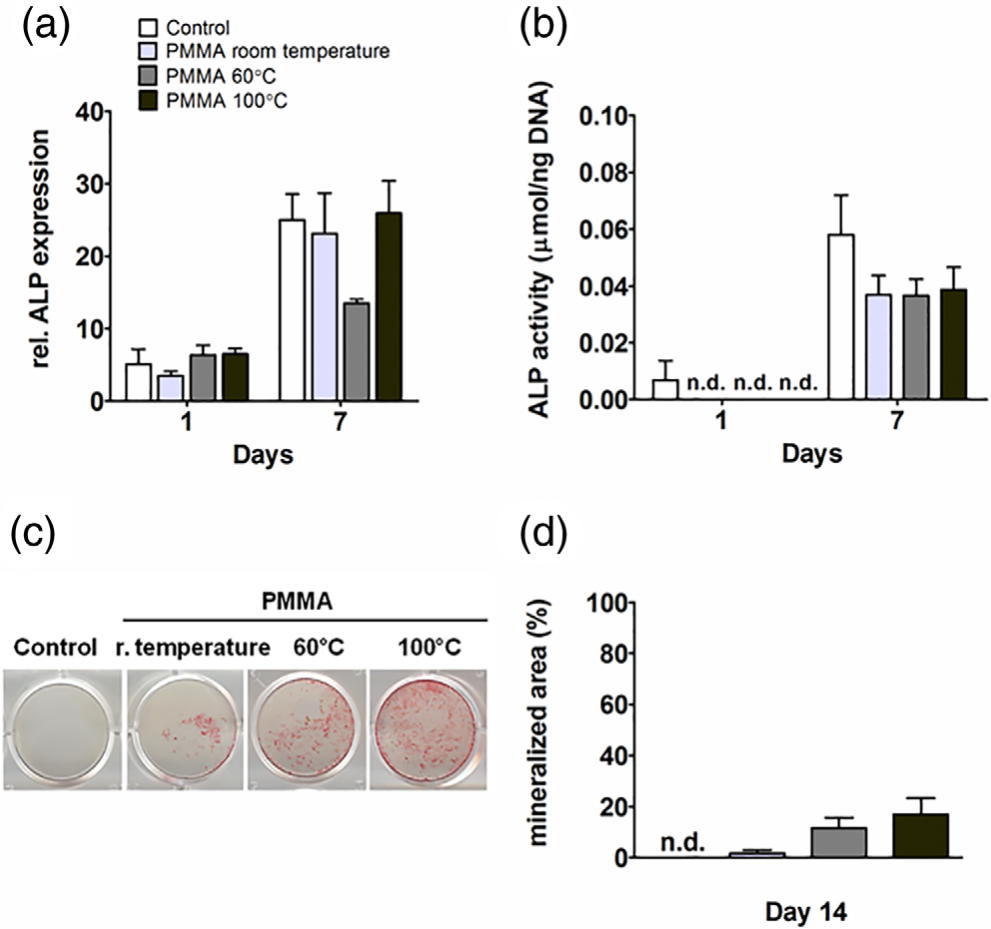
Effect of polymethyl methacrylate (PMMA) on ALP gene expression, ALP activity, and mineralization in human osteoblasts (hOBs). hOBs were cultured with PMMA polymerized at room temperature, at 60°C, or at 100°C. (a) ALP gene expression at day 1 and 7. (b) ALP activity at day 1 and 7. (c) Mineralization at day 14. (d) Quantification of mineralized matrix at day 14. Values are mean ± SEM of duplicate cultures from three independent experiments (*n* = 3) using bone cells obtained from two donors. *Significant effect of PMMA, *p* < .05

In hOBs, PMMA did not inhibit ALP gene expression at day 1 and 7, neither ALP activity at day 7 (Figure 6a,b). Mineral deposition was enhanced in hOBs cultured with PMMA, polymerized at distinct temperatures at day 14 (Figure 6c). Quantification of mineralized matrix did not show increased mineral deposition by hOBs cultured with PMMA compared to controls where mineralization was low or not detected (Figure 6d).

### 3.4 Markers of osteoblast‐to‐osteoclast communication

**Figure 7:**
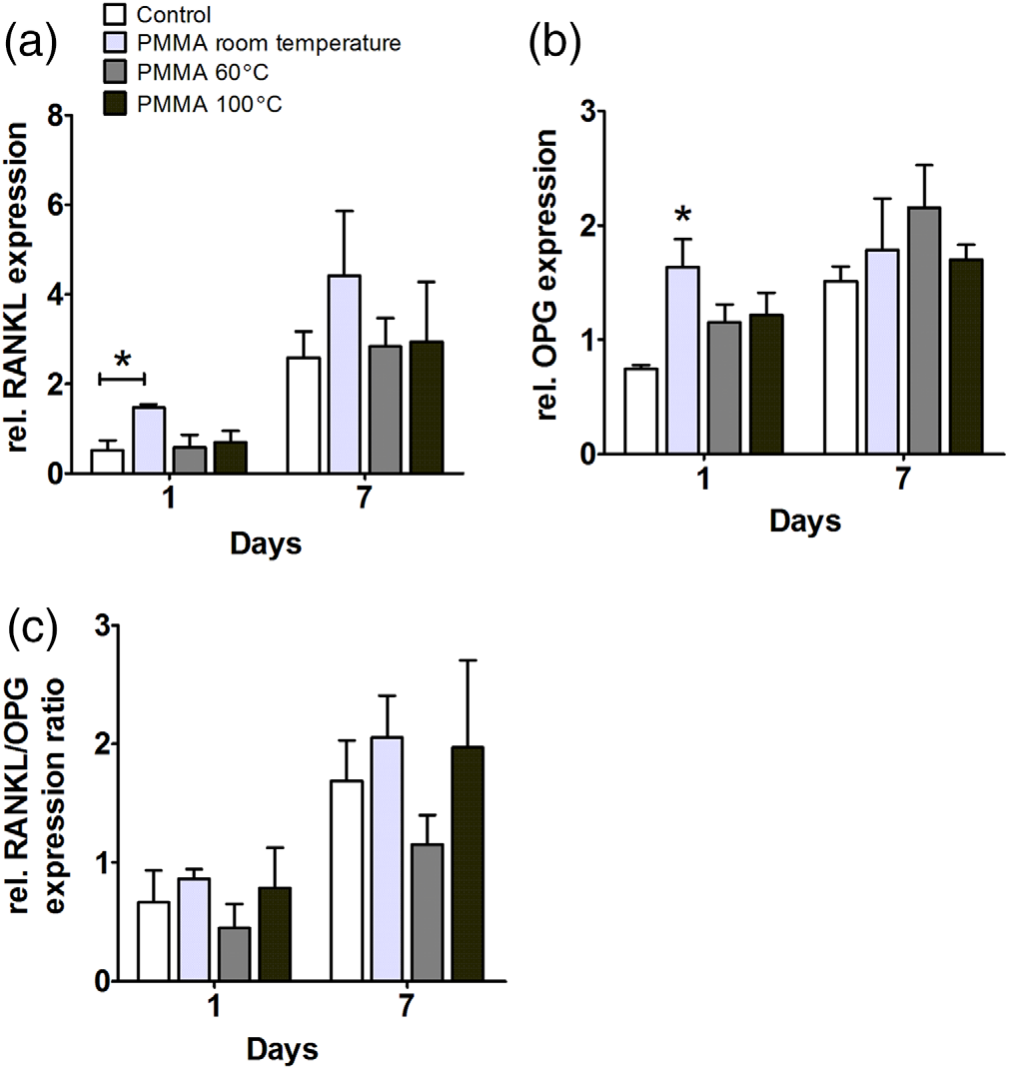
Effect of polymethyl methacrylate (PMMA) on *RANKL* and *OPG* gene *expression in* human adipose stem cells (hASCs). hASCs were cultured with PMMA polymerized at room temperature, at 60°C, or at 100°C. (a) RANKL gene expression at day 1 and 7. (b) OPG gene expression at day 1 and 7. (c) *RANKL/OPG* ratio at day 1 and 7. Values are mean ± SEM of duplicate cultures from three independent experiments (*n* = 3) using hASCs obtained from five donors. *Significant effect of PMMA, *p* < .05

To study the effect of PMMA, polymerized at distinct temperatures, on osteoclast‐activation markers by hASCs or hOBs, gene expression analysis of RANKL and OPG was performed. An increased RANKL/OPG ratio is considered to favor bone resorption (Lacey et al., 1998). PMMA polymerized at room temperature enhanced RANKL expression by hASCs by 2.9‐fold at day 1. There were no significant differences in RANKL gene expression by hASCs between the groups at day 7 (Figure 7a). PMMA polymerized at room temperature enhanced OPG gene expression by 2.2‐fold in hASCs at day 1. PMMA, polymerized at distinct temperatures, did not affect OPG gene expression by hASCs at day 7 (Figure 7b). PMMA did not increase the RANKL/OPG ratio at day 1 and 7 (Figure 7c).

**Figure 8:**
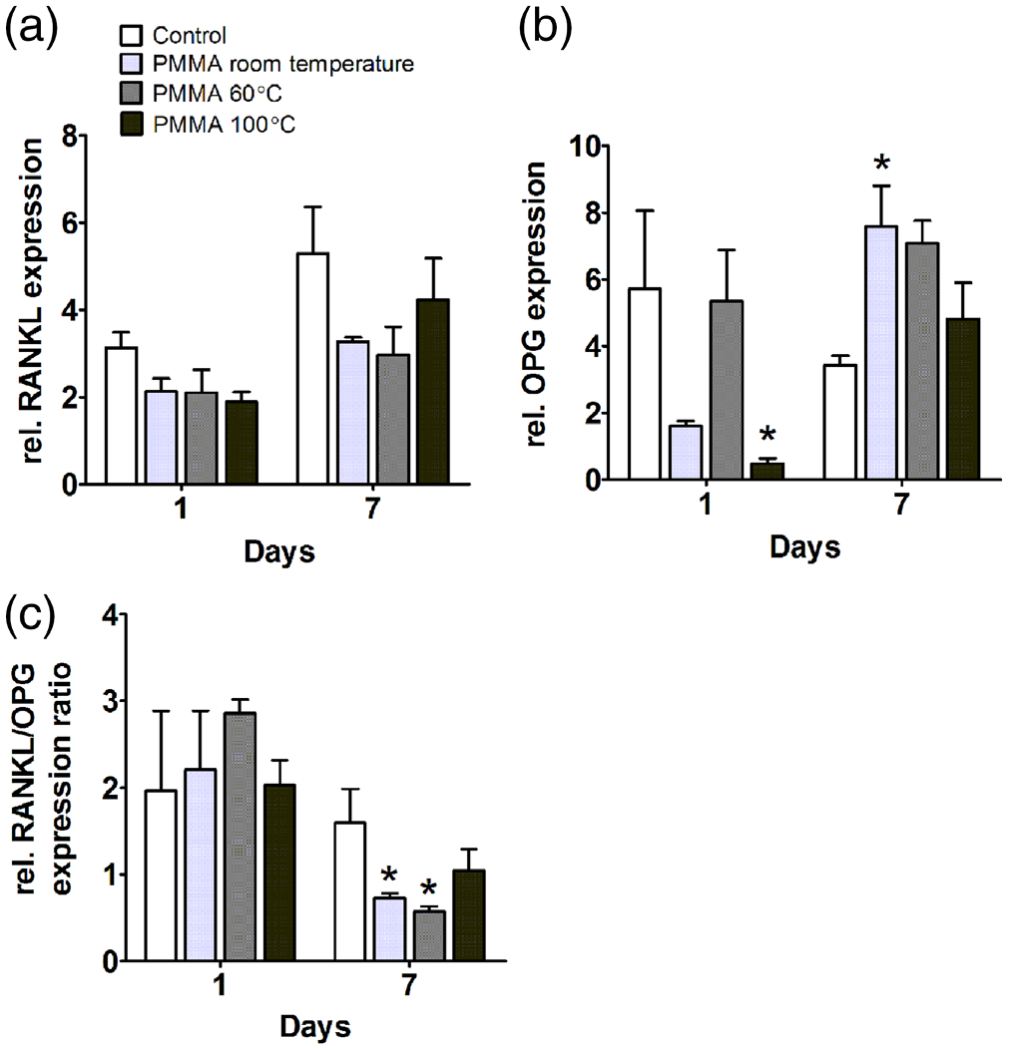
Effect of polymethyl methacrylate (PMMA) on *RANKL* and *OPG* gene expression in human osteoblasts (hOBs). hOBs were cultured with PMMA polymerized at room temperature, at 60°C, or at 100°C. (a) *RANKL* gene expression at day 1 and 7. (b) OPG gene expression of at day 1 and 7. (c) RANKL/OPG ratio at day 1 and 7. Values are mean ± SEM of duplicate cultures from three independent experiments (*n* = 3) using bone cells obtained from two donors. *Significant effect of PMMA, *p* < .05

In hOBs, PMMA, polymerized at distinct temperatures, did not affect RANKL gene expression at day 1 and 7 (Figure 8a). PMMA polymerized at 100°C decreased OPG gene expression by 11‐fold at day 1 while PMMA polymerized at room temperature enhanced OPG expression by twofold at day 7 (Figure 8b). PMMA polymerized at room temperature, and at 60°C decreased the RANKL/OPG ratio at day 7 by 2.1‐fold and by 2.7‐fold, respectively (Figure 8c).

## 4 DISCUSSION

Custom‐made PMMA bone cement is frequently used in craniomaxillofacial surgery for the treatment of large cranial defects due to its high mechanical strength and stability. Despite positive clinical outcomes, there is still ongoing debate whether the potential release of non‐reacted monomers by PMMA cause adverse, cytotoxic effects on the bone tissue surrounding the implanted PMMA (Belkoff & Molloy, 2003; Dahl et al., 1994; Urrutia, Bono, Mery, & Rojas, 2008). MSCs recruited to the implant site, and osteoblasts in the surrounding bone tissue may be affected by the release of non‐reacted monomers. Thus, MSCs and osteoblasts are highly relevant for the in vitro evaluation of possible cytotoxic effects of PMMA. We hypothesized that PMMA is cytotoxic by releasing non‐reacted monomers thereby adversely affecting metabolic activity, proliferation, osteogenic and osteoclast activation potential by human hMSCs and hOBs. We found that PMMA did not cause a biological relevant reduction in viability, metabolic activity, proliferation, and expression of osteogenic differentiation markers in hASCs and hOB. PMMA also did not enhance expression of osteoclast‐activation markers in hASCs that were grown in the presence of discs of PMMA, polymerized at distinct temperatures, in order to manipulate the potential release of non‐reacted monomers.

In our experiments we included TheraCal LC® as an internal positive control for monomer cytotoxicity. TheraCal LC® contains hydrophilic monomers such as HEMA and PEGDMA. These hydrophilic monomers have been reported to reduce cell viability and to induce cell cycle perturbation in peripheral blood mononuclear cells obtained from healthy and HEMA‐sensitized patients, and in murine RAW cells in a dose‐dependent manner (Chang et al., 2005; Orimoto et al., 2013). Our results showing that TheraCal LC® increased LDH activity and decreased metabolic activity and DNA content in hASCs and hOBs at day 1 and 7 agree with these studies. The use of TheraCal LC*®* as a positive control for cytotoxicity strengthens our findings that PMMA is not cytotoxic for MSCs and hOBs, as it shows that our assays are sensitive enough to measure adverse effects of monomers.

Polymerization temperature and time considerably affect the residual monomer content of polymers. Increasing the polymerization temperature in denture base resins from 30 to 60°C has been shown to decrease residual monomer of the polymer (Vallittu et al., 1998). In addition, when heat‐cured denture base resins polymerized at 70°C are allowed to polymerize for an additional period at 100°C, then the concentration of residual monomers of the polymer are significantly reduced when compared with a resin polymerized at 70°C only (Vallittu et al., 1998). Thus, increasing the polymerization temperature of PMMA has been suggested as an option to improve the biocompatibility of PMMA for clinical purposes. In our study we used PMMA, polymerized at distinct temperatures and for different time periods, that is, PMMA was polymerized at room temperature during 15 min, at 60°C during 5 min, or at 100°C during 20 min. PMMA bone cement polymerized at all three temperatures decreased DNA content in hOBs, but not hASCs, at day 1, suggesting that PMMA may delay early proliferation of osteoblasts, but not MSCs, and that heat treatment has no effect, suggesting that leaching of monomers may not be the cause for the decrease in cell number. Addition of PMMA, polymerized at distinct temperatures, did not increase LDH levels, nor did it decrease metabolic activity in hASCs and hOBs, indicating that PMMA did not exert cytotoxic effects on these cells. This is in accordance with findings by others, showing that different PMMA bone cements do not affect viability of hMSCs cultured with or without osteogenic differentiation medium (Pauksch, Hartmann, Szalay, Alt, & Lips, 2014). In contrast, PMMA without a post‐polymerization step exerts cytotoxic effects on mouse fibroblasts (L929), which was counter‐acted when PMMA was post‐polymerized in a water bath at 55°C for 60 min. This suggests that duration of polymerization at a specific temperature might affect cytotoxicity caused by PMMA in some cell types, but not in hOBs and hASCs.

Since MSCs and osteoblasts respond to chemotactic signals released after placement of an implant material in vivo, we analyzed whether PMMA affected the expression of osteogenic differentiation markers by hASCs, and the expression of osteogenic lineage markers in hOBs. We found that PMMA, polymerized at distinct temperatures, did not inhibit the expression of RUNX2, COL1, osteocalcin, ALP activity or matrix mineralization in hASCs or hOBs, which are specific indicators of bone formation. PMMA polymerized at room temperature decreased RUNX2 gene expression in hOBs but only at day 7, without affecting other osteogenic markers. Thus, our findings show that hASCs differentiate into osteogenic cells, and that hOBs maintain their osteogenic properties even in the presence of PMMA. This is in accordance with findings by others showing that PMMA does not decrease osteogenic differentiation of human bone marrow MSCs (BM*MSCs*) cultured with or without osteogenic differentiation medium after 21 days (Pauksch et al., 2014). Taken together, our findings suggest that PMMA is unlikely to interfere with the osteogenic differentiation potential of MSCs recruited at an implant site, and osteoblasts present in the bone tissue surrounding the PMMA implanted.

PMMA wear particles have been reported to increase bone resorption, resulting in aseptic loosening of PMMA in total joint arthroplasties (Amstutz, Campbell, Kossovsky, & Clarke, 1992). Murine bone marrow cultures incubated with PMMA particles show that RANKL expression is an essential cytokine mediator of PMMA particle‐induced bone resorption (Clohisy, Frazier, Hirayama, & Abu‐Amer, 2003). RANKL is expressed in osteoblasts, and activates osteoclasts resulting in enhanced bone resorption, while osteoprotegerin (OPG) is an antagonistic endogenous receptor that upon binding with RANKL inhibits osteoclastogenesis (Kang et al., 2014). The study of RANKL, RANK, and OPG is highly relevant for clinical applications, because PMMA may trigger the release of bone‐resorbing factors at the implant‐bone interface, resulting in the failure of the implant (Ayre, Denyer, & Evans, 2014). PMMA polymerized at room temperature enhanced the expression of RANKL as well as OPG in hASCs at day 1. The increased expression of RANKL and OPG did not result in differences in RANKL/OPG ratio, which indicates that production of osteoclasts factors which promote osteoclast activation are not enhanced by PMMA. PMMA polymerized at 100°C decreased OPG expression in hOB only at day 1, which did not result in differences in RANKL/OPG ratio. PMMA polymerized at room temperature enhanced OPG gene expression, and similar to PMMA polymerized at 60°C, it decreased the RANKL/OPG ratio in hOB, indicating that osteoclast activation potential may be decreased. Thus, PMMA does not appear to induce osteoclast activation potential by hOBs and hASCs, and therefore PMMA is unlikely to enhance bone resorption at the implant site, *although further* in *vivo studies* using PMMA *should be perform*ed to unequivocally demonstrate that PMMA do not induce bone resorption.

The present study has some limitations. The measurement of the osteogenic differentiation markers was performed at the level of mRNA expression rather than protein. However, the results of gene expression of osteogenic markers were in agreement with the results of ALP activity measurement and bone nodule formation, strengthening the conclusion that PMMA does not affect osteogenic differentiation of hOBs or hASCs. Although we did not perform osteoclast culture and bone resorption assays in our experiments, we analyzed the expression of RANKL and OPG in hASCs and hOBs. RANKL and OPG are widely acknowledged as key regulators of osteoclastogenesis (Kobayashi, Udagawa, & Takahashi, 2009). The study of the effect of the exothermic reaction during PMMA bone cement is highly relevant and therefore future studies should address this issue. The study of the exothermic reaction during PMMA polymerization was out of the scope in our study.

In conclusion, our results show that PMMA is not cytotoxic, and does not interfere with the osteogenic differentiation potential of hASCs and hOBs, even when polymerization of the material occurs at distinct temperatures. In addition, PMMA does not enhance production of osteoclast regulatory markers by hASCs and hOBs in vitro. Thus, in contrast to our hypothesis, our data suggests that PMMA, polymerized at distinct temperatures, does not inhibit bone formation. Therefore, these data support the notion that PMMA bone cement is suitable for the treatment of critical size cranial defects.

## CONFLICT OF INTEREST

The authors declare no potential conflict of interest.
